# Niraparib with Abiraterone Acetate and Prednisone for Metastatic Castration-Resistant Prostate Cancer: Phase II QUEST Study Results

**DOI:** 10.1093/oncolo/oyad008

**Published:** 2023-03-30

**Authors:** Kim N Chi, Neil Fleshner, Vincenzo Emanuele Chiuri, Siska Van Bruwaene, Jason Hafron, Douglas G McNeel, Peter De Porre, Raymond Scott Maul, Mahesh Daksh, Xiaogang Zhong, Gary E Mason, Ronald F Tutrone

**Affiliations:** University of British Columbia, BC Cancer – Vancouver Center, Vancouver, BC, Canada; Princess Margaret Cancer Centre, University Health Network, Toronto, ON, Canada; I. Veris Delli Ponti Hospital, Scorrano, Lecce, Italy; Department of Urology, AZ Groeninge Hospital, Kortrijk, Belgium; Michigan Institute of Urology, West Bloomfield, MI, USA; University of Wisconsin Carbone Cancer Center, Madison, WI, USA; Janssen Research & Development, Beerse, Belgium; Janssen Research & Development, Los Angeles, CA, USA; Janssen Research & Development, Raritan, NJ, USA; Janssen Research & Development, College Park, MD, USA; Janssen Research & Development, Spring House, PA, USA; Chesapeake Urology Research Associates, Towson, MD, USA

**Keywords:** castration-resistant prostate cancer, PARP inhibitor, homologous recombination repair, niraparib, abiraterone acetate

## Abstract

Niraparib (NIRA) is a highly selective inhibitor of poly (adenosine diphosphate-ribose) polymerase, PARP1 and PARP2, which play a role in DNA repair. The phase II QUEST study evaluated NIRA combinations in patients with metastatic castration-resistant prostate cancer who were positive for homologous recombination repair gene alterations and had progressed on 1 prior line of novel androgen receptor-targeted therapy. Results from the combination of NIRA with abiraterone acetate plus prednisone, which disrupts androgen axis signaling through inhibition of CYP17, showed promising efficacy and a manageable safety profile in this patient population.

## Teaser

The QUEST study evaluated niraparib in combination with other agents in separate cohorts of patients with metastatic castration-resistant prostate cancer and alterations in genes associated with homologous recombination repair. This article reports on the safety and efficacy of the combination of niraparib with abiraterone acetate plus prednisone.

## Introduction

Up to approximately 30% of patients with metastatic castration-resistant prostate cancer (mCRPC) harbor alterations in genes associated with homologous recombination repair (HRR), rendering them susceptible to poly (adenosine diphosphate-ribose) polymerase (PARP) inhibition.^1,2^ In addition, PARP1 has been found to regulate both androgen receptor (AR) function and response to DNA damage. Niraparib (NIRA), a potent and highly selective inhibitor of PARP1 and PARP2, is approved in the USA, Canada, Europe, and China for use in adult patients for several indications, including ovarian, fallopian tube, and primary peritoneal cancer^3-6^ and is currently under study for the treatment of prostate cancer. The AR axis remains an important oncogenic driver and therapeutic target for mCRPC.^1^ Therefore, targeting both oncogenic dependencies may result in improved outcomes in prostate cancer.^1,7,8^ This QUEST study (ClinicalTrials.gov Identifier: NCT03431350) is a phase II, multicenter, open-label clinical trial designed to evaluate NIRA in combination with other agents in separate cohorts of patients with mCRPC and alterations in genes associated with HRR. We report on the safety and efficacy of the combination of NIRA with abiraterone acetate plus prednisone (AAP).

## Methods

### Patients

Patients with mCRPC who were biomarker-positive for an alteration in genes associated with HRR (*ATM*, *BRCA1*, *BRCA2*, *BRIP1*, *CHEK2*, *FANCA*, *HDAC2*, and *PALB2*) by either blood or tissue assay (*HDAC2* only by blood assay) and who had progressed on 1 prior line of novel AR-targeted therapy for mCRPC were eligible. Prior treatment with taxane-based therapy and AR-targeted therapy outside of the mCRPC setting was allowed. All patients provided written informed consent.

### Trial Design and Interventions

This was an open-label, single-arm, single-stage, and phase II study. Patients received NIRA as two 100 mg capsules (200 mg total), abiraterone acetate as four 250 mg tablets (1000 mg total) once daily, and prednisone as 5 mg tablets twice daily (10 mg total).

### Assessments

The primary endpoints were composite response rate (CRR; evaluated in the intent-to-treat [ITT] efficacy population) and frequency and severity of adverse events (AEs; evaluated in the safety population). CRR is defined as the proportion of patients with ≥1 of the following: objective radiographic response in subjects with measurable disease, overall circulating tumor cell (CTC) response, or prostate-specific antigen decline ≥50% (PSA50). Overall CTC response is defined as a patient with CTC0 response at 8 weeks (baseline CTC per 7.5 mL of blood >0 and 8 weeks post-baseline CTC = 0) or CTC conversion (baseline CTC per 7.5 mL of blood ≥5 and post-baseline CTC <5 with a confirmation CTC <5 taken ≥4 weeks later). Key secondary endpoints were overall CTC response rate, objective response rate (ORR) (per RECIST 1.1), and radiographic progression-free survival (rPFS).

### Statistical Analysis

For the ITT population, 2-sided 90% CIs were calculated for CRR, ORR, and overall CTC response. rPFS was evaluated using the Kaplan–Meier method.

## Results

### Patient Characteristics

Twenty-four patients were included in the safety analysis, of whom 1 was excluded from the ITT population (found to be HRR negative); 17 patients had *BRCA2* alterations, 2 had *ATM*, 2 had *CHEK2*, 1 had *FANCA*, and 1 had *PALB2*. Of the total safety population, with a median age of 73 years, 15 (62.5%) patients had a Gleason score of ≥8 at the initial diagnosis. Twenty-two (91.7%) patients had skeletal metastases, 9 (37.5%) had lymph node metastases, and 1 had liver metastases at baseline. All ITT patients had received ≥1 prior therapy for prostate cancer, and all patients had received a prior next-generation AR inhibitor (Supplementary Table S1). The median duration of NIRA + AAP treatment was 10.3 months (range, 0.7-22.0). With a median follow-up of 18 months, 8 patients remained on treatment at the analysis cut-off.

### Efficacy

In the ITT population, CRR was 56.5% (90% CI, 37.5-74.2; *n* = 13). There were 10 patients who had measurable disease; 5 reached partial response, 2 had stable disease, and 3 had progressive disease as their best response. ORR was 50% (90% CI, 9.0-40.4), and the median duration of response was 4.7 months (range, 3.7-8.2). Responses occurred in patients with *BRCA* and other HRR gene alterations. Overall CTC response rate was 26.1% (90% CI, 12.0-45.1; *n* = 6), and 7 (30.4%; 90% CI, 15.2-49.6) patients reached PSA50 response (Table 1). CTC0 response was observed in 4 (17.4%; 90% CI, 6.2-35.5) patients, and CTC conversion was observed in 5 (21.7%; 90% CI, 9.0-40.4) patients. Median rPFS was 11.0 months (90% CI, 9.7-not estimable). Event-free survival rates for 6 and 12 months were 74.1% and 46.7%, respectively.

**Table 1.** CRR in the ITT population.

		NIRA + AAP	
	Measurable disease at baseline	No measurable disease at baseline	Total
Analysis set: ITT, *N*	10	13	23
Patients with composite response			
No. of patients with composite response	7	6	13
CRR	70.0%	46.2%	56.5%
90% CI	(39.3-91.3)	(22.4-71.3)	(37.5-74.2)
Patients with objective response^a^			
No. of patients with objective response	5	N/A	5
ORR	50.0%	N/A	N/A
90% CI	(22.2-77.8)	N/A	N/A
Patients with overall CTC response^b^			
No. of patients with overall CTC response	2	4	6
Overall CTC response rate	20.0%	30.8%	26.1%
90% CI	(3.7-50.7)	(11.3-57.3)	(12.0-45.1)
Patients with PSA50			
No. of patients achieving PSA50	3	4	7
PSA50 response rate	30.0%	30.8%	30.4%
90% CI	(8.7-60.7)	(11.3-57.3)	(15.2-49.6)

^a^Only patients with measurable disease were included in the total ORR calculation.

^b^Overall CTC response = CTC response at 8 weeks or CTC conversion.

Abbreviations: CRR, composite response rate; CTC, circulating tumor cell; ITT, intent-to-treat; N/A, not applicable; NIRA + AAP, niraparib 200 mg, abiraterone acetate 1000 mg, and prednisone 10 mg; ORR, objective response rate; PSA50, prostate-specific antigen decline ≥50%.

### Safety

Common grade 1/2 AEs included constipation, fatigue, nausea, vomiting, and decreased appetite. The most common grade 3 AEs were anemia (41.7%), thrombocytopenia (20.8%), fatigue (16.7%), and neutropenia (12.5%; Table 2). Treatment-emergent AEs (TEAEs) were managed with dose interruption, reduction, or both, as well as supportive care, including transfusion. TEAEs led to dose interruption in 11 patients and dose reduction in 9 patients, the most common reasons for interruption/reduction were anemia (*n* = 7), thrombocytopenia (*n* = 5), and neutropenia (*n* = 3). TEAEs led to discontinuation in 2 patients (thrombocytopenia [*n* = 1]; thrombocytopenia and anemia [*n* = 1]). There were serious drug-related AEs in 3 patients (1 each with lower gastrointestinal hemorrhage, asthenia and noncardiac chest pain, and anemia) and no deaths because of AEs.

**Table 2.** Treatment-emergent adverse events by grade (≥15% of patients).

TEAEs, *n* (%)	Grade 1/2 (*N* = 24)	Grade 3/4^a^ (*N* = 24)
Anemia	3 (12.5)	10 (41.7)
Fatigue	9 (37.5)	4 (16.7)
Constipation	12 (50)	0
Thrombocytopenia	5 (20.8)	5 (20.9)
Nausea	9 (37.5)	0
Vomiting	8 (33.3)	0
Decreased appetite	8 (33.3)	0
Neutropenia	2 (8.4)	3 (12.5)
Back pain	5 (20.8)	0
Dyspnea	3 (12.5)	1 (4.2)
Dizziness	4 (16.7)	0
Weight decreased	4 (16.6)	0

^a^Only two grade 4 AEs, each in 1 patient: thrombocytopenia and pulmonary embolism.

## Discussion

The results presented suggest NIRA + AAP has promising efficacy and a manageable toxicity profile in patients with mCRPC and alterations in genes associated with HRR who had progressed on 1 prior line of novel AR-targeted therapy. The current phase II study is limited by the open-label, single-arm trial design, and the small patient population. Whereas these findings are consistent with the BEDIVERE^9^ study, further data are needed to assess the efficacy and safety of this combination. Two ongoing phase III studies evaluate NIRA + AAP versus placebo + AAP in patients with mCRPC^2^ and metastatic castration-sensitive prostate cancer.^10^ For MAGNITUDE,^2^ the primary analysis showed a statistically significant and clinically meaningful improvement for rPFS with NIRA + AAP for patients with BRCA1/2 alterations (HR = 0.533 [95% CI, 0.361-0.789; 2-sided *P* = .0014]) as well as the combined HRR gene altered population (HR = 0.729 [95% CI, 0.556-0.956; *P* = .0217]).

## Supplementary Material

oyad008_suppl_Supplementary_Table_1

## Data Availability

The data sharing policy of Janssen Pharmaceutical Companies of Johnson & Johnson is available at https://www.janssen.com/clinical-trials/transparency. As noted on this site, requests for access to the study data can be submitted through Yale Open Data Access [YODA] Project site at http://yoda.yale.edu.
