# Supplementary material for: Niraparib with Abiraterone Acetate and Prednisone for Metastatic Castration-Resistant Prostate Cancer: Phase II QUEST Study Results
Source: Oncologist. 2023 Mar 30;28(5):e309–12. doi: 10.1093/oncolo/oyad008 (PMC10166146; doi:10.1093/oncolo/oyad008)
Supplement: oyad008_suppl_Supplementary_Table_1 [file oyad008_suppl_supplementary_table_1.docx]

**Supplementary Table. Baseline demographics and disease characteristics.**

|  | Total |
| --- | --- |
| Characteristic | N = 24 |
|  |  |
| Age, years |  |
| Median | 73.0 |
| Range | (58-88) |
| Race, n (%) |  |
| Black or African American | 3 (12.5) |
| White | 21 (87.5) |
| Ethnicity, n (%) |  |
| Hispanic or Latino | 1 (4.2) |
| Not Hispanic or Latino | 23 (95.8) |
| Gleason score at initial diagnosis, n (%) |  |
| <7 | 2 (8.3) |
| 7 | 5 (20.8) |
| ≥8 | 15 (62.5) |
| Unknown | 2 (8.3) |
| Extent of disease, n (%) |  |
| Bone | 22 (91.7) |
| Bone only | 13 (54.2) |
| Liver | 1 (4.2) |
| Lymph node | 9 (37.5) |
| Other^a^ | 2 (8.3) |
| ECOG PS score, n (%) |  |
| 0 | 14 (58.3) |
| 1 | 10 (41.7) |
| Prior taxanes, n | 7 |
| Prior AR-targeted therapies, n | 24 |
| Any prostate cancer–related radiotherapy, n | 24 |
| Any prostate cancer–related surgery, n | 24 |

ECOG PS, Eastern Cooperative Oncology Group performance status.

^a^Other includes 1 patient with lymph node lesions and 1 patient with a left prostate bed lesion.
